# A Qualitative Study of Perceptions and Preferences Regarding Social and Behavioral Risk Screening Among Primary Care Patients

**DOI:** 10.1007/s11606-023-08344-8

**Published:** 2023-08-14

**Authors:** Sae Takada, Zewei Shen, Philippe Bourgois, O. Kenrik Duru, Lillian Gelberg, Maria Han, Marjan Javanbakht, Steve Shoptaw, Kenneth Wells, Gery Ryan

**Affiliations:** 1 Division of General Internal Medicine and Health Services Research, Department of Medicine, David Geffen School of Medicine, University of California, Los Angeles, CA USA; 2 VA Greater Los Angeles Health System, Los Angeles, CA USA; 3 Center for Social Medicine and Humanities, Semel Institute for Neuroscience and Human Behavior, Department of Psychiatry and Biobehavioral Sciences, David Geffen School of Medicine, University of California, Los Angeles, CA USA; 4 Department of Anthropology, University of California, Los Angeles, CA USA; 5 Department of Family Medicine, David Geffen School of Medicine, University of California, Los Angeles, CA USA; 6 Office of Health Care Transformation and Innovation, VA Greater Los Angeles Health System, Los Angeles, CA USA; 7 Department of Health Policy and Management, Fielding School of Public Health, University of California, Los Angeles, CA USA; 8 Department of Epidemiology, Fielding School of Public Health, University of California, Los Angeles, CA USA; 9 Department of Psychiatry and Biobehavioral Sciences, David Geffen School of Medicine, University of California, Los Angeles, CA USA; 10 Center for Health Services and Society, Semel Institute for Neuroscience and Human Behavior, Department of Psychiatry and Biobehavioral Sciences, David Geffen School of Medicine, University of California, Los Angeles, CA USA; 11 Kaiser Permanente Bernard J. Tyson School of Medicine, Pasadena, CA USA

## Abstract

**Background:**

Despite its relevance for healthcare settings, social and behavioral risk screening is not systematically performed by clinicians or healthcare systems.

**Objective:**

To address clinician concerns, such as social and behavioral risk screening disrupting the clinician-patient relationship and lack of resources to respond, we interviewed primary care patients at an academic medical center regarding their perceptions and preferences on social and behavioral risk screening.

**Participants:**

Between September and December 2020, we recruited a convenience sample of 14 English-speaking primary care patients 18 years + from three clinics affiliated with an academic medical center.

**Approach:**

Using a semi-structured interview guide, we asked about the importance of social and behavioral risk screening, whether or not and how to share social and behavioral risk factors, and how social and behavioral risk factors are addressed. We used a multi-step analytic process to identify the range and commonality of participants’ responses thematically.

**Key Results:**

Participants recognized that social and behavioral risk factor domains were relevant to primary care and important for treating the patient as a whole person. Participants preferred a conversation regarding social and behavioral risk factor with their primary care providers (PCPs), and suggested that, if surveys are used, they be followed with an open-ended, in-person discussion. Participants also suggested framing the discussion as something that is done routinely with all patients so that patients do not feel judged. Participants felt comfortable sharing social and behavioral risk factors when they trusted their PCPs, and felt that discussing social and behavioral risk factors with their PCPs built trust. Participants recognized that resources exist outside of the clinic, and suggested that PCPs distribute lists of relevant community resources to patients.

**Conclusion:**

In our study of primary care patients on perceptions and preferences about screening and addressing social and behavioral risk factors, we found that patients were willing to share social and behavioral risk factors with their PCP, preferred an in-person discussions with or without a survey, and wanted a list of community resources to address their needs.

## Introduction

In the USA, healthcare systems are increasingly incorporating social and behavioral risk factor screening into care.^1^ These efforts are motivated by strong evidence that social and behavioral risk factors shape health, policy statements by National Academy of Medicine,^2^ American Academy of Pediatrics,^3^ and American Academy of Family Physicians^4^ calling for integration of social and behavioral risk factors into clinical care, and financial incentives for clinicians and health systems to improve health outcomes and reduce cost of care.^5–8^ Early studies have demonstrated the relevance and feasibility of social and behavioral risk factor screening in healthcare settings. A multi-center survey among clinicians showed that over 80% agreed that social and behavioral risk factors were an issue for most patients, and that screening should be part of medical care.^9^ Implementation of social and behavioral risk screening in a range of clinical settings showed that screening and referral using electronic medical record (EMR) workflows are feasible,^10^ that many patients endorse at least one domain^10–13^ and desire assistance in meeting their needs,^12^ and that screening may lead to interventions that reduce health care utilization.^7,12^

Despite evidence of its relevance and feasibility, social and behavioral risk screening is not consistently performed by clinicians or systematically implemented by healthcare systems.^9,14^ In addition to practical barriers to implementing social and behavioral risk screening such as lack of time,^9,14^ training and expertise,^11,14^ and concerns for data entry and other administrative burdens,^11,13^ clinicians express concern about how screening would change the patient experience, such as whether the screening may disrupt the clinician-patient relationship^9,11,15^ or make patients feel uncomfortable or stigmatized.^9,16^ Clinicians also note absence of resources to remediate social and behavioral risk factors,^9,13,15^ making them wonder whether the healthcare system is the appropriate place to screen and engage with remediation of social and behavioral risk factors.^13,17^

To explore these important concerns, we conducted an exploratory study (similar to the Exploration phase in the Exploration, Preparation, Implementation, Sustainment framework^18^) with the primary objective to understand how patients perceived social and behavioral risk screening. We interviewed primary care patients at a high-volume, urban academic medical center that had recently integrated a social and behavioral risk screening tool in its EMR, adapted from domains and questions recommended by the National Academy of Medicine in 2014.^2^ We asked about whether patients wanted to share social and behavioral risk factors with their primary care providers (PCPs), their perceptions of importance of social and behavioral risk screening to primary care, preferences on how to share social and behavioral risk factors with their PCP, and preferences on how social and behavioral risk factors are addressed in primary care.

## Methods

### Setting and Patient Population

The goal of this exploratory study^19^ is to illuminate the fundamental range of responses patients have to social and behavioral risk factor screening in primary care in order to assess whether such screening would be acceptable. The study took place in an urban, academic medical center with over 50 primary clinics across Southern California^20^ that had recently introduced a social and behavioral risk screening module into the EMR in November 2019.^21^ The module was developed for the Epic EMR system based on domains and measures selected by a committee of experts convened by the National Academy of Medicine in 2014, who were tasked to identify core domains to be included in EMRs and recommend specific measures under each domain.^2,22^ The subsequent Epic EMR module included ten domains: depression, smoking, alcohol use, physical activity, stress, financial strain, food insecurity, transportation needs, social connections, and intimate partner violence. The National Academy of Medicine committee and the EPIC module called these domains “social determinants of health.”^2,22^ Since then, increasing interest to integrate social factors into health care has led to more nuanced discussions of these concepts, such as the distinctions between “social determinants” (the conditions in which people are born, grow, live, work, and age, which are shaped by the distribution of resources), “social risk factors” (the specific adverse social conditions that are associated with poor health), and “behavioral risk factors” (unhealthy behaviors such as smoking, alcohol use, and lack of physical activity).^23^ Going forward, we will use the term “social and behavioral risk factors” to refer to the ten domains included in the module.

At the time of the study, screening for some domains were systematically integrated into clinical care while others were not. Even prior to the integration of the screening module in November of 2019, depression, smoking, and alcohol use were routinely screened by medical assistants and licensed vocational nurses in primary care clinics. For example, unpublished analyses of EMR data showed that, between November 2017 and June 2021, 75% of patients were screened at least once for depression, 99% for smoking, and 91% for alcohol use. On the other hand, other domains were rarely screened after implementation of the module. The same EMR data showed that 1% were screened for financial strain, 0.5% for transportation needs, 0.5% for food insecurity, 0.5% for social connections, and 1% for intimate partner violence. The low screening rates are similar to those at another institution with EMR-based tools before they were integrated into the clinical workflow.^24^

We recruited English-speaking primary care patients 18 years or older from three primary care clinics affiliated with the academic medical center. A primary care patient was defined as a patient who had at least one primary care encounter in the previous 24 months. We chose primary care patients, as primary care settings could potentially play an important role in shaping how social and behavioral risk factors are screened and addressed within healthcare settings.^13^ Our objective was to understand the range of perspectives on social and behavioral risk screening. We were most interested in themes that occurred frequently as well as rare ones that would have important implications on how we conduct screening. We estimated that we would reach this point after interviewing 15 to 20 participants. As in-person recruitment was restricted during the COVID-19 pandemic, we used the patient portal to message randomly selected primary care patients regarding the study. Of the 39,351 eligible primary care patients in the three clinics, a total of 424 patients were messaged in weekly batches over the course of 5 weeks, and 47 expressed interest in participating. We consented and scheduled 17 whom we were able to reach by telephone and agreed to participate. Two of those scheduled did not present for the interview and could not be reached for rescheduling, and one was not eligible due to receiving primary care at another institution. We recruited while we conducted interviews, and ST, ZS, and GR met regularly to discuss findings. We discontinued recruitment when we had reached thematic saturation, i.e., we were no longer encountering new, major themes. Participants provided verbal informed consent for audiorecording and transcription. The institutional review board at the University of California, Los Angeles, approved this research.

### Data Collection

We developed a 45 to 60-min semi-structured interview guide (see Appendix) informed by the literature to explore patients’ perceptions and preferences of social and behavioral risk screening in primary care. We opened the interview by introducing the concept of social and behavioral risk, and showed the ten domains that are currently included in the EMR module (depression, smoking, alcohol use, physical activity, stress, financial strain, food insecurity, transportation needs, social connections, intimate partner violence). For each of the ten domains, we asked about whether they would want to share it with their PCP (“Would you want to share this information with your provider?”), their perceptions of importance of screening to primary care (“How important do you think it is for your provider to know this information?”), preferences on how they share it with their PCP (“What do you think is the best way for you to share this information with your provider?”), and preferences on how it is addressed in primary care (“What do you think is the best way to address this issue?”). The interviews were conducted by authors ST and ZS by telephone or videoconference between October and December 2020. Participants were compensated with a $50 gift certificate for their time. Interviews were professionally transcribed.

### Data Analysis

To identify the range and commonality of themes mentioned by participants, we used a multi-step iterative analytic process.^25^ Drawing on what authors ST and ZS learned while conducting the interviews and taking notes as well as regular team meetings to discuss emerging areas of interest, we began by creating an initial codebook in which we categorized patient quotes into three main areas based on our interview guide in a deductive coding approach: importance of social and behavioral risk screening in primary care, preferences for how to share social and behavioral risk factors in primary care, and expectations for addressing social and behavioral risk factors in primary care. Then, authors ST and ZS read each interview transcript to extract core quotes and independently sorted them into groups of similar quotes to identify subthemes using an inductive coding approach. We addressed coding discrepancies through discussion and consensus. Finally, ST, ZS, and GR discussed and reached consensus on key subthemes. We used Dedoose (version 9.0.46) qualitative data management software to facilitate the analysis. Illustrative quotes are listed in Table 1.

**Table 1:** Additional Illustrative Quotes from Participant Interviews

Theme	Subtheme	Quotes
Relevance of social and behavioral risk factors	Social and behavioral risk factors affect health	“If the doctor doesn’t know that you smoke and you have hypertension or any other condition, it’s a matter of life and death.” (Smoking)
		“Alcohol can have such consequences for one’s body, one’s health, and one’s cognition and one’s well-being and social behaviors.” (Alcohol use)
		“[Financial strain] does affect your wellbeing, just your stress levels and if financially you don’t have enough to eat that’s going to affect your health.” (Financial strain)
		“Because not having transportation to make it to your doctor is very stressful indeed. I’ve been in that situation. I missed a lot of doctor’s appointments early in my career, and it was very problematic.” (Transportation need)
		“If [a patient] had a decent amount of social connection and now that’s dropped, for whatever reason, illness, divorce, whatever, that person now might be a bit more apt to be stressed and depressed versus if he had people and outlets to reach out to.” (Social connections)
	PCPs should know the patient as a whole person	“Since your primary care physician is your first line of defense or care, I think it’s important that they know what’s going on with you.” (All domains)
Sharing social and behavioral risk factors	Prefer in-person discussion with PCP	“Forms are helpful for eliminating a lot of questions, but I’d rather have the questions asked in person. I’d rather have a personal conversation about it.” (All domains)
	Role of survey tool in social and behavioral risk factor screening	“It could be done where you just do a self-administered form first and then the healthcare provider looks that over. The important piece, though, is it should then be reviewed in real-time with you with the healthcare provider.” (All domains)
	Open-ended approach	“I would ask a general question first and then if they didn’t really get more specific, then ask the second and third question. [For example,] ‘In the world of COVID, how are you dealing with it, how are you handling the stress? What are the things that you do to deal with it?’” (All domains)
	Importance of framing the discussion	“Preface it that these are issues that a lot of people are dealing with and can really impact health and wellbeing, so that’s the reason we are trying to discuss them with our patients to make sure we’re addressing your needs as best we can” (All domains)
	Trust in PCP	“A doctor is somebody that you have to confidence in as a person and you have to be able to talk about things that aren’t necessarily obviously medical problems. And so, you want to be able to have a sense of trust and the sense that the doctor knows you as a person and not just as a set of measurements of different components of your blood and so on.” (All domains)
Addressing social and behavioral risk factors	Does not expect PCP to address social and behavioral risk factors	“I’m not sure that’s his area. Doctors, they took all types of chemistry and physics and science courses, they didn’t take a lot of accounting, finance, tax and investment courses, so I don't think they have the expertise to give you that kind of advice.” (Food insecurity)
	Wants PCP to acknowledge social and behavioral risk factors	“I was just sharing with her about the frustrations I’ve had with my weight gain over this coronavirus and how I seem to put everybody first except myself and just her acknowledgement of that was really—she’s so awesome as far as listening and acknowledging it.” (Stress)
	Wants PCP to consider social and behavioral risk factors when making recommendations	“Talking about what we do to relieve stress. I usually meditate and do Qigong. Well, I haven’t been doing it the last few weeks. So it would be, ‘Well, why are you not doing it? What are the things you usually do? If you’re doing them now, great. If you’re not doing it, what do you need to do to get yourself back to doing it?’” (Stress)
	Wants to receive a list of community resources	“It would be great, like, if they had resources to refer you to that might even be outside the healthcare system. Like, ‘Oh, here’s a resource list of how you can get assistance with transportation or how you could get assistance with food or whatever the thing might be.’ The things that are out in the community.” (All domains)
		“I think it’s always more helpful if something’s tailored, but I also don’t know how realistic that is. I think in general, from the patient point of view, you get a very generic pamphlet and that’s sometimes not as helpful or not as attractive. Patients might pay less close attention to something that seems really generic.” (All domains)
	Wants in-person case management	“Have an on-call somebody who works in the doctor’s office, who can come in and sit down with you and say, ‘I see that you indicated you’ve been having some domestic problems with violence. Would you like to talk about that with me?’” (Intimate partner violence)

## Results

We interviewed six female participants and eight male participants. Seven of our participants identified as White, one identified as Hispanic/Latino,  one identified as African American/Black, two identified as European, and three identified as other race or had no response. This was similar to the racial and ethnic identities of primary care patients in this academic medical center, which are approximately 50% White non-Hispanic, 5% Black non-Hispanic, 10% Hispanic all races, 10% Asian non-Hispanic, and 10% other.^26^ The mean age of our participants was 67 (SD 11). Our analyses found the following patient-respondent themes: (1) all domains were important and relevant to primary care, and part of knowing the patient as a whole person; (2) preference for a conversation regarding social and behavioral risk factors with their PCPs, and surveys be followed with an in-person discussion; (3) sharing social and behavioral risk factors requires trusting their PCP, and discussing social and behavioral risk factors builds trust; (4) varying levels of addressing social and behavioral risk factors are acceptable, including a community resource guide to give out to patients.

### Social and Behavioral Risk Screening is Whole Person Care

Most participants recognized that all social and behavioral risk factor domains were important and relevant to primary care: “I think all of these issues affect our health and wellbeing for sure.” Some participants made a distinction between social and behavioral risk factors that have direct and indirect impacts on health. Participants associated alcohol use, smoking, physical activity, food insecurity, and stress with specific health problems, such as cardiovascular disease. For example, one participant stated, “You need physical activity to be alive. Diabetes, heart disease, you have a whole list of problems if you don’t take care of yourself. So, it’s important that the doctor understands how much exercise you’re doing because if you’re not doing exercise, at least he can give you medications to help you.” In contrast, participants described how certain domains, such as social connections and transportation needs, impact health problems indirectly, often through creating stress. For example, one participant noted, “Transportation needs and financial strain would lead to the stress, so those would be more indirect. Social connections is more indirect and to a certain extent depression is indirect as well, compared to something like smoking and alcohol and physical activity and stress.” Some participants felt that it was more important to discuss social and behavioral risk factors that have direct impacts on health with their PCP than those that have indirect impacts. Participants also noted that transportation needs and financial strain lead to problems with access to care, such as missed appointments and medications.

More generally, many participants believed that knowing about a patient’s social and behavioral risk factors is part of knowing the patient as a whole person, and would allow PCPs to make better medical decisions. One participant stated, “The doctor should know everything about you because you’re depending on them to make decisions about your health. So, they have to know what’s going on with you, otherwise they can’t make a good decision.”

### Open-Ended Conversations Rather Than Solely Surveys

Regarding social and behavioral risk screening, participants generally preferred a conversation with their PCP to discuss risk factors rather than fill out a survey. Some participants expressed frustration that they have filled out surveys in the waiting room but did not have them addressed by their PCPs during the visit. However, other participants noted that a survey has some advantages. It may be more time-efficient by eliminating topics irrelevant to the patient or highlighting topics the patient would like to discuss in-depth with the PCP during the visit. One participant stated, “If I didn’t see a specific list or wasn’t asked specific questions it might not occur to me to bring up financial stress or social connections or maybe even the [intimate partner] violence at an appointment.” Also, participants believed that patients may be more likely to disclose stigmatized or traumatic topics on a survey, such as food insecurity or intimate partner violence: “It would be easier to write that down or acknowledge it, because they could be in denial themselves about it. If it’s on paper, it might be easier to even just put a little thing that maybe there’s something going on.” Therefore, participants stated that a survey tool could supplement or improve the in-person discussion.

Regarding how a discussion about social and behavioral risk factors should take place, participants preferred that the PCP engage in open-ended conversation rather than ask directed questions. For example, one participant explained:

When you talk to people, they don’t ask you a series of questions that are not related directly to you. So, I would think that it’s better for the doctor to avoid that sort of checklist approach and to get to know the patient, talk to the patient, and gradually realize what the patient’s needs are and what kind of problems he or she brings up. That would make the relationship more natural.

Another participant suggested using open-ended questions followed by probing questions: ‘What kind of car do you drive? Or how’s your car doing?’ Little things like that. I think if you ask open-ended questions then you get a better picture for what’s really going on. You just can’t come out and ask, because people will get defensive.”

Participants also suggested using a preamble to explain that all patients are asked these questions so the patient does not feel judged or offended. One participant suggested the following statement: “As your doctor, I want to see you as a whole person and these questions help me understand some of the factors going on in your life so I can help you more or get you the help you need.”

### Sharing Social and Behavioral Risk Factors Builds Trust for PCP

Participants stated that they need to have built a trusting relationship with their PCPs in order to be willing to share social and behavioral risk factors, particularly depression, alcohol use, financial strain, and intimate partner violence. For example, one participant stated regarding sharing financial strain, “I trust her and I feel comfortable with her. I don't feel like I need to hide things from her or I don’t feel like I should be ashamed.” Some participants explained how they had waited until they had a relationship with their PCPs before sharing social and behavioral risk factors: “The first visit was, we checked the numbers, we made sure we got the referrals and the tests and everything done. And then, when I went back and felt comfortable, I was more willing to share.”

Participants also stated that discussing social and behavioral risk factors with their PCPs builds trust in the patient-clinician relationship: “It helps build a trusting relationship with your physician if they care about you.” Finally, participants mentioned that the PCP would get know their social and behavioral risk factors through the natural course of the patient-clinician relationship.

### Varying Expectations for Addressing Social and Behavioral Risk Factors

A few participants stated that they did not expect PCPs to address some of the social and behavioral risk factors as they were outside of the PCPs’ expertise, such as social connections, food insecurity, and financial strain: “In some of these cases, it’s hard to say what the doctor can do.” Some participants expressed the value of the PCP in listening to their challenges: “Just her acknowledgement of stress was really—she’s so awesome as far as listening and acknowledging it.” Many participants thought that the PCP could take social and behavioral risk factors into consideration when making clinical decisions and or providing counseling. One participant stated, “If it were a financial strain issue and it was an issue of affording care, then I would hope that she’d be able to take that into consideration and make some recommendations.” For some social and behavioral risk factors, such as depression, stress, or social connections, participants thought that a referral to a mental health specialist within the health system would be appropriate.

Participants commonly noted that there are resources outside of the health system to address social and behavioral risk factors, such as food banks, substance use treatment programs, hotlines, and benefits such as Supplemental Nutrition Assistance Program (SNAP) and transportation services from insurance providers. However, they thought that it is unrealistic for their PCPs to know about local community resources, and suggested that PCPs have a list of community resources to give out to their patients. For example, a participant stated:

Not everyone knows what’s out there for people and it’s difficult to know everything you know to be a physician and know about financial opportunities out there. But it seems to me [the medical center] can give some of the resources to our physicians so they have those resources there. They have a sheet that tells them where they can refer people if they’re having issues with food insecurity, transportation needs, financial strains, stuff like that.

Some participants suggested having a generic list of local resources, so that patients who are reluctant to share social and behavioral risk factors with their PCPs can still take advantage of the resources. Other participants suggested that a generic list could be overwhelming, and suggested that the PCP highlight relevant items: “I just don’t know how practical it would be to have resource lists be highly, highly tailored, but if somebody brings something to you and they circle the things that they think might be particularly relevant to your situation.”

Finally, a few participants suggested having a member of the care team who could provide in-person case management services and follow up with the patient, but thought that it may not be feasible in the current healthcare system. One participant stated, “In an ideal world, you could have a personal case worker-type person that was going to follow up with you, but that could be a little bit idealistic because I don't know if there are enough people or resources to have that kind of personalized attention for each patient.”

## Discussion

In this qualitative exploratory study of screening and addressing social and behavioral risk factors among primary care patients at an academic medical center, we found that patients are willing to discuss social and behavioral risk factors with the PCPs, preferred open-ended conversations about them with their PCPs, and accepted a wide range of ways in which their social and behavioral risk factors are addressed. Our findings showed that patients perceived two seemingly dichotomous roles of the primary care provider—to listen to patients’ values and priorities, and to address the social, economic, and political structures that shape patients’ health^27–29^—as important aspects of building a trusting relationship and making medical decisions together.

Most participants were willing to share social and behavioral risk factors with their PCP because they believed these were relevant to health and more generally reflected “what’s going on” with the patient, both of which were important in the patient and PCP making decisions together about care. This is consistent with findings from prior studies of social and behavioral risk screening which found that patients believed that screening was appropriate^30^ and important^31^ because those risk factors were relevant to health.

Patients strongly preferred having an open-ended conversation about social and behavioral risk factors with their PCPs rather than, or in conjunction with, a “checklist approach” of filling out a survey or answering directed questions. This is in contrast to how research and implementation of social and behavioral screening has centered around the use of survey tools, with or without integration into the EMR.^2,32^ Participants saw social and behavioral risk screening as an integral part of their PCPs getting to know them and building a human relationship; participants discussed social and behavioral risk factors with their PCPs because participants trusted their PCPs, and, in turn, felt that the discussion of these issues built trust. The extensive literature on trust in physicians identifies dimensions of trust such as dependability^33,34^ competence,^33–35^ confidentiality,^33^ honesty,^34^ and communication,^35^ but not much has been examined regarding building trust in physicians through the discussion of life circumstances. Because trust in physicians has been associated with various health outcomes,^36^ discussion of social and behavioral risk factors could have unintended benefits.

At the same time, participants acknowledged that a survey may be more efficient, better for sensitive topics, and could help remind them of what they want to discuss in person. Therefore, a two-step process of a self-administered survey tool followed by in-person discussion with the PCP was acceptable to many participants. To make the discussion less threatening and stigmatizing, participants noted the importance of framing the discussion as something that is done routinely with all patients in order to see them as a whole person and to provide better care.

Finally, consistent with prior research,^31^ participants expressed a range of expectations on how social and behavioral risk factors are addressed: that the PCPs acknowledge their challenges, take social and behavioral risk factors into consideration when making recommendations, and make internal referrals to mental health specialists when appropriate. Most participants expected a generic list of local resources that could be tailored to their particular needs. This is consistent with two studies on food insecurity screening in which patients preferred a list of local resources.^37,38^ Developing and maintaining a list of community resources is resource-intensive,^39^ and health care systems may benefit from partnering with organizations that maintain resources and facilitating referrals.^40^ The few who requested availability of case management for referral and follow-up admitted that it may not be feasible in the current health care system. While clinicians and administrators may express reluctance to implement screening without capacity for in-person referral and linkage to community-based resources,^15,39^ our findings support preliminary evidence that resource handouts may be as acceptable as in-person assistance.^41^

These findings were shared with medical center stakeholders with the goal to implement social and behavioral risk screening that is feasible and acceptable for clinicians, patients, and the health system. Other ongoing quality improvement efforts at the medical center to integrate social and behavioral risk factors into care include developing a survey to assess social needs among primary care patients and expanding social work support in primary care clinics.

We acknowledge several limitations to this study. This was an exploratory study with a convenience sample of 14 patients, with the goal to understand the range of responses to social and behavioral risk screening and the degree to which the responses were shared among participants. Although we do not know the precise rates at which patients may hold these perceptions and preferences, we know that these are commonly held among a significant number of patients. The next step would be to conduct a systematic, closed-ended survey of primary care patients. The participants were English-speaking primary care patients affiliated with an urban academic medical center, and were 50% White, similar to the patient population served by this medical center. The findings may not be generalizable to other populations or settings serving a more diverse patient population. Participants were not selected for having experience with social needs. However, a prior study found that there was no difference in food insecurity screening preference between patients by food insecurity status.^38^

In conclusion, in our exploratory study of primary care patients on their perceptions and preferences regarding screening and addressing social and behavioral risk factors, we found that patients were willing to share social and behavioral risk factors with their PCP, preferred an in-person discussion (with or without out a survey), and desired a list of community resources to address their needs. Participants felt comfortable sharing social and behavioral risk factors because they trusted their PCP, and felt that discussing social and behavioral risk factors also built trust with their PCP. Clinicians may want to broach social and behavioral risk screening with patients as an opportunity to strengthen the clinician-patient relationship rather than assuming it makes patients uncomfortable or stigmatized. Clinicians and patients may benefit if a preamble explains the purpose of screening prior to administration of a survey screening tool, and follow up immediately with an in-person, open-ended discussion. Finally, clinicians should be aware that patients are open to accepting varying degrees of social and behavioral interventions, including acknowledgement and counseling, and are aware of the limitations of resources in the healthcare setting but may appreciate efforts at social and behavioral risk screening and linkage to services.

## Data Availability

The data that support the findings of this study are available on request from the corresponding author. The data are not publicly available due to privacy restrictions.

## Appendix. Interview Guide

I am going to ask you questions about social determinants of health. Social determinants of health are behaviors and living situations that impact people’s health.

There are 10 social determinants of health included in the electronic medical records. Some of them affect why people get sick, and some of them affect how they get care. They are: depression, tobacco smoking, alcohol use, physical activity, stress, financial insecurity, food strain, transportation needs, social connections, and intimate partner violence.

- 1. Would you want to share this information with your health care provider? Have you shared this information with your health care provider?
- 2. How important do you think it is for health care provider to know this information?
- 3. What do you think is the best way for you to share this information to your health care provider? What do you think is the best way for you feel most comfortable in saying what is really going on?
- 4. What do you think is the best way to address this issue?

## References

[1] Gottlieb LM, Wing H, Adler NE (2017). A Systematic Review of Interventions on Patients’ Social and Economic Needs. Am J Prevent Med.

[2] Institute of Medicine (2014). Capturing Social and Behavioral Domains and Measures in Electronic Health Records: Phase 2.

[3] **Hagan JF, Shaw JS, Duncan PM.** Bright futures guidelines for health supervision of infants, children, and adolescents : pocket guide. In: Joseph F. Hagan, Jr., Judith S. Shaw, Paula M. Duncan, eds. Fourth edition. ed. Elk Grove Village, Illinois: American Academy of Pediatrics; 2017.

[4] American Academy of Family Physicians. AAFP Committed to Addressing the Social Determinants of Health, Striving for Health Equity. https://www.aafp.org/news/media-center/kits/social-determinants-of-health.html. Accessed 18 Jun 2023

[5] **Bachrach DP, H., Wallis, K. Lipson M.** Addressing Patients' Social Needs: An Emerging Business Case for Provider Investment: The Commonwealth Fund 2014. https://www.commonwealthfund.org/publications/fundreports/2014/may/addressing-patients-social-needs-emerging-business-case-provider. Accessed 18 Jun 2023.

[6] Alley DE, Asomugha CN, Conway PH, Sanghavi DM (2016). Accountable Health Communities — Addressing Social Needs through Medicare and Medicaid. New England J Med.

[7] Centers for Medicare and Medicaid Services. Making the Business Case for Addressing Health-Related Social Needs 2022. https://innovation.cms.gov/media/document/ahc-reading-hosp-spotlight. Accessed 18 Jun 2023.

[8] Centers for Medicare and Medicaid Services. Accountable health communities model. 2019. https://innovation.cms.gov/initiatives/ahcm/. Accessed 18 Jun 2023.

[9] Schickedanz A, Hamity C, Rogers A, Sharp AL, Jackson A (2019). Clinician Experiences and Attitudes Regarding Screening for Social Determinants of Health in a Large Integrated Health System. Med Care..

[10] Buitron de la Vega P, Losi S, Sprague Martinez L, Bovell-Ammon A, Garg A, James T (2019). Implementing an EHR-based Screening and Referral System to Address Social Determinants of Health in Primary Care. Med Care.

[11] Gold R, Bunce A, Cowburn S, Dambrun K, Dearing M, Middendorf M (2018). Adoption of Social Determinants of Health EHR Tools by Community Health Centers. Ann Family Med.

[12] Schickedanz A, Sharp A, Hu YR, Shah NR, Adams JL, Francis D (2019). Impact of Social Needs Navigation on Utilization among High Utilizers in a Large Integrated Health System: a Quasi-experimental Study. J Gen Intern Med.

[13] Tong ST, Liaw WR, Kashiri PL, Pecsok J, Rozman J, Bazemore AW (2018). Clinician Experiences with Screening for Social Needs in Primary Care. J Am Board Fam Med..

[14] Fraze TK, Brewster AL, Lewis VA, Beidler LB, Murray GF, Colla CH (2019). Prevalence of Screening for Food Insecurity, Housing Instability, Utility Needs, Transportation Needs, and Interpersonal Violence by US Physician Practices and Hospitals. JAMA Netw Open..

[15] Garg A, Boynton-Jarrett R, Dworkin PH (2016). Avoiding the Unintended Consequences of Screening for Social Determinants of Health. JAMA..

[16] Bloch G, Rozmovits L, Giambrone B (2011). Barriers to primary care responsiveness to poverty as a risk factor for health. BMC Family Pract.

[17] Maani N, Galea S (2020). The Role of Physicians in Addressing Social Determinants of Health. JAMA.

[18] Moullin JC, Dickson KS, Stadnick NA, Rabin B, Aarons GA (2019). Systematic review of the Exploration, Preparation, Implementation, Sustainment (EPIS) framework. Implement Sci.

[19] Arain M, Campbell MJ, Cooper CL, Lancaster GA (2010). What is a pilot or feasibility study? A review of current practice and editorial policy. BMC Med Res Methodol.

[20] UCLA Health. Primary Care. 2023. https://www.uclahealth.org/medical-services/primary-care#:~:text=Our%20network%20includes%20more%20than,provide%20primary%20care%20to%20children. Accessed 18 Jun 2023

[21] Gold R, Cottrell E, Bunce A, Middendorf M, Hollombe C, Cowburn S (2017). Developing Electronic Health Record (EHR) Strategies Related to Health Center Patients' Social Determinants of Health. J Am Board Fam Med.

[22] Adler NE, Stead WW (2015). Patients in Context — EHR Capture of Social and Behavioral Determinants of Health. New England J Med.

[23] Alderwick H, Gottlieb LM (2019). Meanings and Misunderstandings: A Social Determinants of Health Lexicon for Health Care Systems. Milbank Q..

[24] Wang M, Pantell MS, Gottlieb LM, Adler-Milstein J (2021). Documentation and review of social determinants of health data in the EHR: measures and associated insights. J Am Med Inform Assoc..

[25] Ryan G, Bernard H (2003). Techniques to Identify Themes. Field Methods - FIELD METHOD..

[26] **Takada S, Chung UY, Bourgois P, Duru KO, Gelberg L, Han M, et al.** Individual and community socioeconomic status and receipt of influenza vaccines among adult primary care patients in a large health care system in Los Angeles, CA. Manuscript under review 2023. 10.1016/j.heliyon.2024.e40476 PMC11625130 39654784

[27] Manelin EB (2020). Health Care Quality Improvement and the Ambiguous Commodity of Care. Med Anthropol Quarterly..

[28] Burson RC, Familusi OO, Clapp JT (2022). Imagining the ‘structural’ in medical education and practice in the United States: A curricular investigation. Soc Sci Med..

[29] Lantz PM, Goldberg DS, Gollust SE (2023). The Perils of Medicalization for Population Health and Health Equity. Milbank Quarterly..

[30] De Marchis EH, Hessler D, Fichtenberg C, Adler N, Byhoff E, Cohen AJ (2019). Part I: A Quantitative Study of Social Risk Screening Acceptability in Patients and Caregivers. Am J Prevent Med.

[31] Byhoff E, De Marchis EH, Hessler D, Fichtenberg C, Adler N, Cohen AJ (2019). Part II: A Qualitative Study of Social Risk Screening Acceptability in Patients and Caregivers. Am J Prevent Med.

[32] Agency for Healthcare Research and Quality. SDOH & Practice Improvement. 2020. https://www.ahrq.gov/sdoh/practice-improvement.html Accessed October 5, 2022.

[33] Anderson LA, Dedrick RF (1990). Development of the Trust in Physician scale: a measure to assess interpersonal trust in patient-physician relationships. Psychol Rep..

[34] Safran DG, Kosinski M, Tarlov AR, Rogers WH, Taira DH, Lieberman N (1998). The Primary Care Assessment Survey: tests of data quality and measurement performance. Med Care..

[35] Greene J, Ramos C (2021). A Mixed Methods Examination of Health Care Provider Behaviors That Build Patients’ Trust. Patient Educ Counsel.

[36] Birkhäuer J, Gaab J, Kossowsky J, Hasler S, Krummenacher P, Werner C (2017). Trust in the health care professional and health outcome: A meta-analysis. PLOS ONE..

[37] **Palakshappa D, Doupnik S, Vasan A, Khan S, Seifu L, Feudtner C, et al.** Suburban Families’ Experience With Food Insecurity Screening in Primary Care Practices. Pediatrics. 2017;140(1). 10.1542/peds.2017-0320 10.1542/peds.2017-0320 28634248

[38] Kopparapu A, Sketas G, Swindle T (2020). Food Insecurity in Primary Care: Patient Perception and Preferences. Fam Med..

[39] **Beidler LB, Razon Na, Lang H, Fraze TK.** “More than just giving them a piece of paper”: Interviews with Primary Care on Social Needs Referrals to Community-Based Organizations. J Gen Intern Med. 2022;37:4160-4167. 10.1007/s11606-022-07531-3 10.1007/s11606-022-07531-3 PMC9708990 35426010

[40] Cartier Y, Fichtenberg C, Gottlieb LM (2020). Implementing Community Resource Referral Technology: Facilitators And Barriers Described By Early Adopters. Health Affairs..

[41] Gottlieb LM, Adler NE, Wing H, Velazquez D, Keeton V, Romero A (2020). Effects of In-Person Assistance vs Personalized Written Resources About Social Services on Household Social Risks and Child and Caregiver Health: A Randomized Clinical Trial. JAMA Network Open..

